# Targeting Aryl Hydrocarbon Receptor Signaling Enhances Type I Interferon-Independent Resistance to Herpes Simplex Virus

**DOI:** 10.1128/Spectrum.00473-21

**Published:** 2021-10-20

**Authors:** Jincheng Chen, Juan Liang, Hui Xu, Wenqi Liu, Shuyan Liu, Lian Duan, Fang Li, Zhaoqin Wang, Yingxia Liu, Brian McSharry, Carl G. Feng, Guoliang Zhang

**Affiliations:** a Savaid Medical School, University of Chinese Academy of Sciences, Beijing, China; b National Clinical Research Center for Infectious Diseases, Guangdong Provincial Clinical Research Center for Tuberculosis, The Third People’s Hospital of Shenzhen, Southern University of Science and Technology, Shenzhen, China; c Biomedical Translational Research Institute, Faculty of Medical Science, Jinan University, Guangzhou, China; d Infections, Immunity and Inflammation, School of Medical Sciences, Faculty of Medicine and Health, The University of Sydney, Sydney, New South Wales, Australia; Barnard College, Columbia University

**Keywords:** aryl hydrocarbon receptor, herpes simplex virus 1, type I interferons, viral replication

## Abstract

The aryl hydrocarbon receptor (AHR) is a ligand-activated transcript factor that plays an important role in regulating immunity and cell differentiation. However, its role in cell-autonomous antiviral resistance has not been fully elucidated. Here, we show that interruption of AHR signaling in human cells by a chemical antagonist or genetic targeting led to significant reductions in the replication of herpes simplex virus 1 (HSV-1) and cytomegalovirus (CMV), revealing an unexpected proviral function of AHR. Interestingly, the enhanced viral control in the absence of AHR is independent of type I interferon (IFN) signaling. Together, these results reveal a previously unknown function of AHR in promoting viral replication *in vitro* and suggest a potential intervention point for treating viral disease.

**IMPORTANCE** This study describes how a virus might utilize host aryl hydrocarbon receptor signaling to promote its replication, even in the presence of type I interferons.

## INTRODUCTION

Herpes simplex virus 1 (HSV-1) is a common pathogen infecting four-fifths of the world’s population (1, 2). After establishing infection in skin and mucosal epithelial cells, the virus can invade neurons (3). HSV-1 infection causes a mild clinical presentation, such as cold sore, when the host provides strong immunity. However, the risk of developing encephalitis and keratitis increases significantly when host immunity declines (4, 5). Although antivirals can mitigate the symptoms in the majority of patients, they fail to completely clear the virus (6). Hence, finding novel therapeutic targets and developing new drugs have important implications in treating HSV-1 infection.

The aryl hydrocarbon receptor (AHR) is a ligand-activated transcript factor, which is activated by a variety of endogenous signaling and exogenous molecules from the diet, microbial flora, metabolites, and environmental pollutants (7, 8). Once activated by ligands, AHR translocates from the cytoplasm to the nucleus and regulates the transcription of numerous gene targets that are involved in the differentiation and proliferation of lymphocytes, macrophages, and dendritic cells (9, 10). AHR immunomodulation function has been studied in a variety of immune-metabolic diseases and inflammatory conditions (11, 12). However, until recently, the impact of AHR on the host’s defense against viral infections has not been fully examined. Several recent studies have shown that the activation of the AHR pathway reduces the virus-specific IgG titer, impairs the differentiation of CD4^+^ and CD8^+^ T cells, and increases inflammation, resulting in increased morbidity and mortality following viral infections (13, 14). Interestingly, the activation of the AHR signaling pathway has been shown to downregulate the type I interferon (IFN)-mediated antiviral response (15). Together, the above-mentioned studies suggested that AHR activation decreases host resistance to virus infection in murine models of viral infection. In this investigation, we demonstrate that AHR also operates in human cells to inhibit cell-autonomous antiviral resistance.

## RESULTS

### Inhibition of AHR increases resistance to HSV-1 infection.

**FIG 1:**
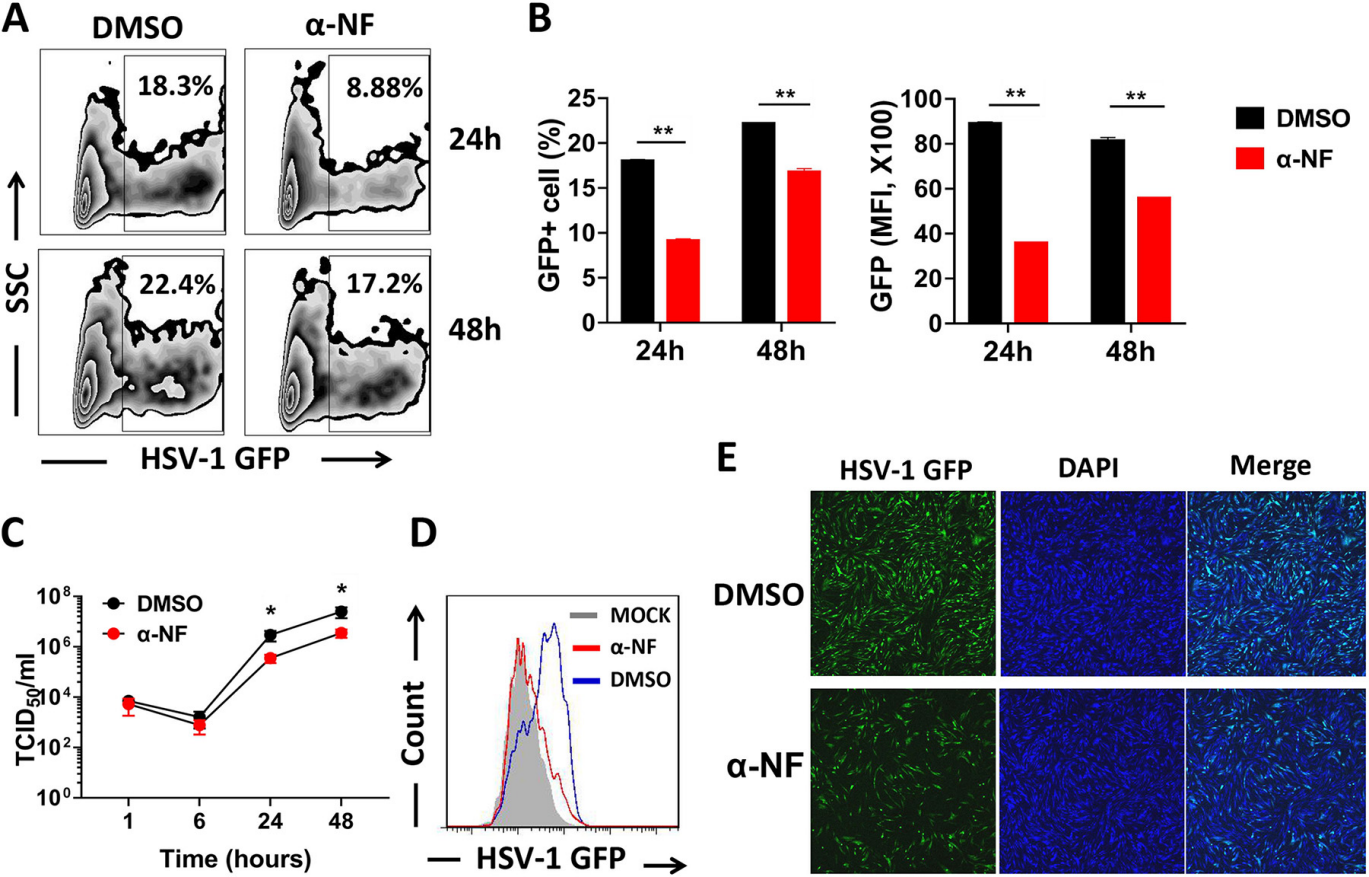
Inhibition of AHR in THP-1 or HFF-1 cells provides resistance to HSV-1 infection. WT THP-1 and HFF-1 cells were pretreated with dimethyl sulfoxide (DMSO) or the AHR antagonist α-NF for 12 h and then infected with GFP-expressing HSV-1. (A) After infection with GFP-expressing HSV-1 in WT THP-1 cells for 24 h and 48 h, GFP^+^ THP-1 cells were determined using flow cytometry. SSC, side scatter. (B) Percentage of GFP-positive cells or mean fluorescence intensity (MFI) shown in a histogram. (C) THP-1 cells were infected with HSV-1 for 1, 6, 24, and 48 h in the absence or presence of α-NF. Cells were lysed, and lysates were serially diluted and then used to infect Vero cells to determine viral titers by a TCID_50_ assay. (D) The count of HSV-1-infected HFF-1 cells was also investigated by flow cytometry. (E) Confocal microscopy was used to visualize HSV-1-infected HFF-1 cells. *, *P* < 0.05; **, *P* < 0.01. Data are shown as means ± SEM.

To elucidate the role of AHR signaling in cell-autonomous resistance to viral infection, we first analyzed AHR expression in untreated and HSV-1-infected human monocytic THP-1 cells and found that the level of the receptor mRNA was elevated following infection (see Fig. S1A in the supplemental material). We next exposed THP-1 cells to green fluorescent protein (GFP)-expressing HSV-1 in the presence or absence of a known AHR antagonist, α-naphthoflavone (α-NF), and GFP expression was determined using flow cytometry 24 h and 48 h later. We observed that antagonizing AHR signaling by α-NF significantly decreased the percentage and intensity of GFP-positive (GFP^+^) THP-1 cells (Fig. 1A and B). To determine whether AHR regulates viral entry or replication, THP-1 cells were infected with GFP-expressing HSV-1 in the presence or absence of α-NF and lysed 1, 6, 24, and 48 h after infection. Viral titers of the cell lysates were determined by a 50% tissue culture infective dose (TCID_50_) assay. We observed that the viral titers in treated and untreated cultures were comparable at 1 h and 6 h. However, by 24 h and 48 h, the former cultures showed significantly reduced viral titers, suggesting that AHR signaling affects the replication rather than the entry of HSV-1 (Fig. 1C). This result was further confirmed by flow cytometry and immunofluorescence imaging in human foreskin fibroblast 1 (HFF-1) cells (Fig. 1D and E).

### *AHR*^−/−^ THP-1 cells are more resistant to HSV-1 infection than *AHR*^+/+^ cells.

**FIG 2:**
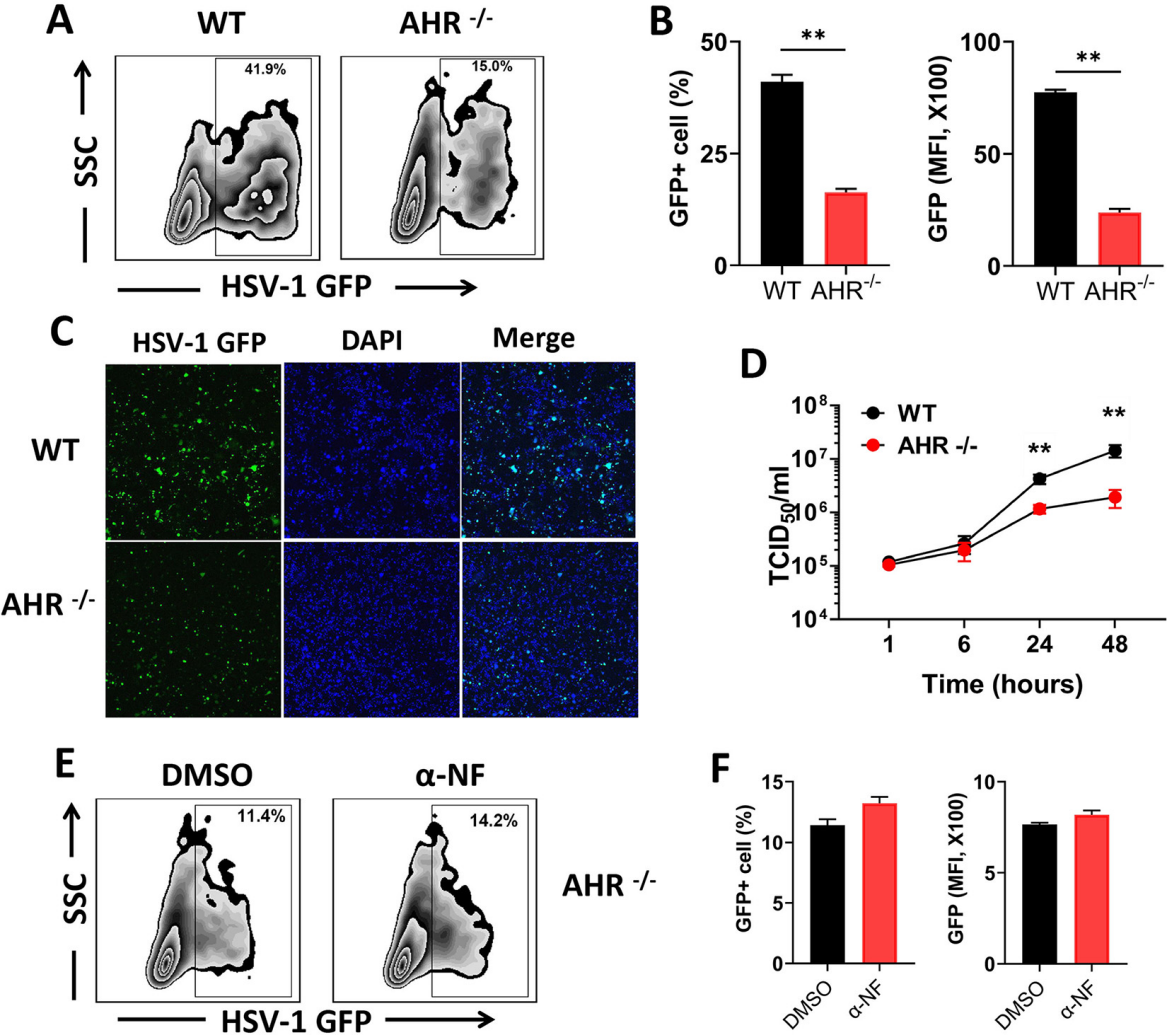
*AHR*^−/−^ THP-1 cells are resistant to HSV-1 infection. WT and *AHR*^−/−^ THP-1 cells were infected with GFP-expressing HSV-1. (A to C) GFP expression at 24 h was determined using flow cytometry (A), and the percentage of GFP-positive cells and mean fluorescence intensity are shown in histograms (B) and confocal microscopy images (C). (D) WT or *AHR*^−/−^ THP-1 cells were infected with HSV-1 for 1, 6, 24, and 48 h. Viral titers were determined by a TCID_50_ assay. (E and F) *AHR*^−/−^ cells were pretreated with DMSO or α-NF and infected with GFP-expressing HSV-1, GFP expression was determined by flow cytometry (E), and the percentage of GFP^+^ cells and the mean fluorescence intensity are shown in histograms (F). *, *P* < 0.05; **, *P* < 0.01. Data are shown as means ± SEM.

To establish the proviral role of AHR more definitively, we generated a stable AHR-deficient THP-1 cell line (*AHR*^−/−^) by employing CRISPR/Cas9 technology. Consistent with the antagonist treatment experiments, the deficiency in AHR signaling in *AHR*^−/−^ cells was associated with a significant reduction in GFP^+^ cells, as demonstrated by both flow cytometry (Fig. 2A and B) and immunofluorescence microscopy (Fig. 2C; Fig. S2), compared to wild-type (WT) THP-1 cells. When the viral titer was determined, we found no differences at the early time points of 1 h and 6 h between WT and *AHR*^−/−^ cells, but the viral titer significantly decreased at 24 h and 48 h in *AHR*^−/−^ cells (Fig. 2D). Interestingly, WT and *AHR*^−/−^ cells treated with the AHR agonist 2,3,7,8-tetrachlorodibenzo-*p*-dioxin (TCDD) showed comparable levels of infection (Fig. S1), an observation consistent with previous studies (14, 16). The lack of a significant increase in viral loads in the agonist-treated cultures could result from the fact that only limited numbers of viral particles can be produced in individual cells.

In addition, the resistance to HSV-1 infection provided by α-NF was abolished in *AHR*^−/−^ cells, which further confirmed that the reduction of HSV-1 infection in *AHR*^−/−^ cells is indeed due to impaired AHR signaling (Fig. 2E and F). We observed that *AHR*^−/−^ cells were also more resistant to GFP-expressing cytomegalovirus (CMV) infection, revealing that the function of AHR in viral replication is not restricted to HSV-1 (Fig. S3). Taken together, these results indicate that AHR signaling enhances viral replication *in vitro*.

### HSV-infected *AHR*^−/−^ THP-1 cells show enhanced IFN-β production.

**FIG 3:**
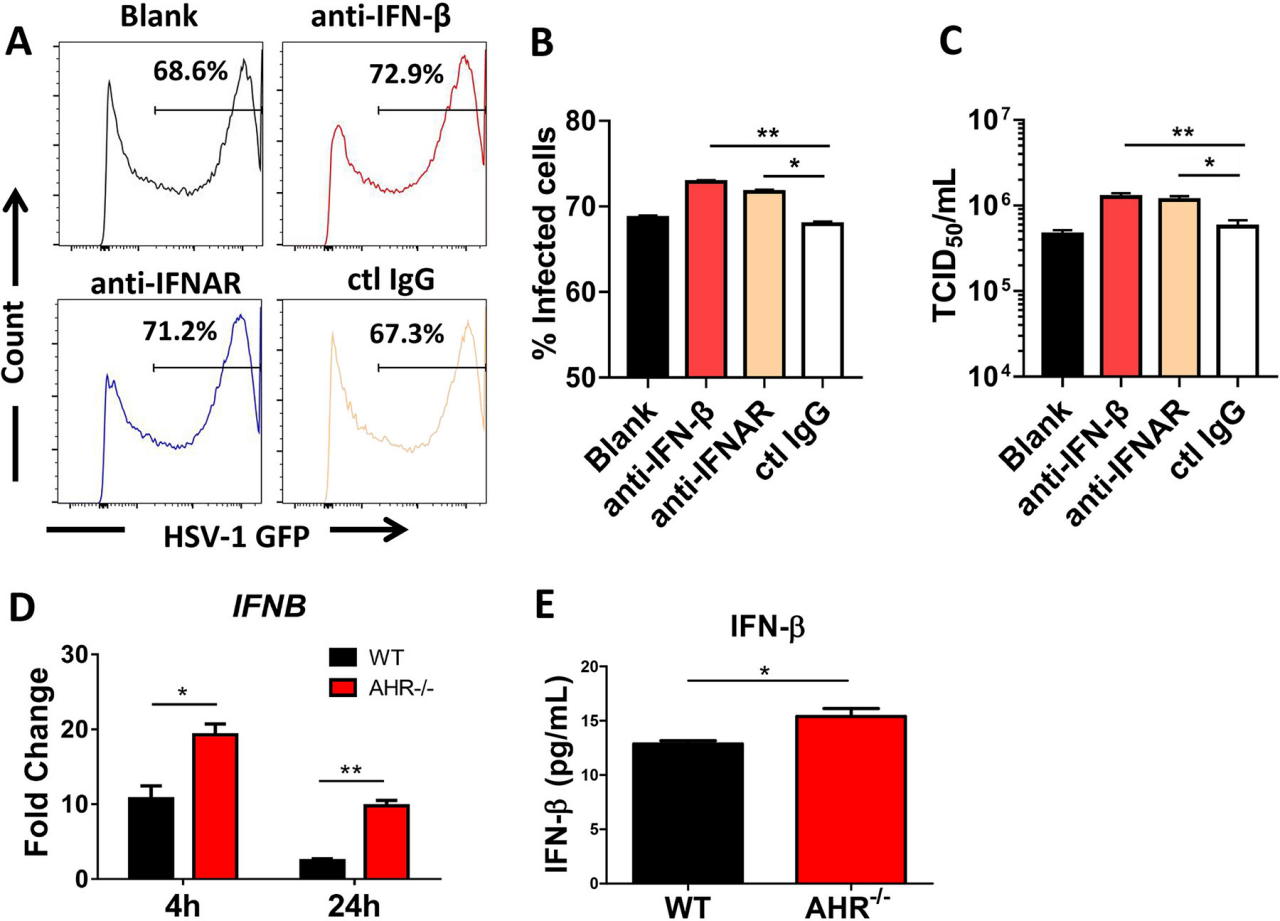
*AHR*^−/−^ THP-1 cells increase HSV-1-induced IFN-β production. (A) WT THP-1 cells treated with IFN-β antibody, IFNAR antibody, the IgG isotype antibody as a control, or an equal volume of medium as a blank were infected with HSV-1 for 24 h, and the percentage of HSV-1-infected cells was measured by flow cytometry. (B) Percentage of GFP-positive cells shown in a histogram. (C) Viral titers in each group were determined by a TCID_50_ assay. (D and E) WT and *AHR*^−/−^ THP-1 cells were infected with HSV-1, mRNA expression was determined by quantitative PCR (qPCR) upon infection for 4 and 24 h (D), and the protein levels of IFN-β were determined by an ELISA after infection for 24 h (E). *, *P* < 0.05; **, *P* < 0.01. Data are shown as means ± SEM.

Recent mouse studies have demonstrated that AHR activation negatively regulated the type I IFN response, in which higher levels of type I IFNs and their inducible genes were observed in AHR-deficient mice and cells (15, 17). In our human cell system, we observed that neutralization of IFN-β or blockade of type I IFN receptor signaling led to a small but significant increase in GFP^+^ cells following HSV-1 infection, as determined by flow cytometric analysis and TCID_50_ assays (Fig. 3A to C). Importantly, we observed that 4 h and 24 h after infection, higher levels of IFN-β mRNA (Fig. 3D) and IFN-β protein (Fig. 3E) were detected in *AHR*^−/−^ cell cultures than in their WT counterparts. Thus far, these results revealed that AHR deficiency promoted IFN-β expression at the mRNA and protein levels.

### Enhanced resistance of *AHR*^−/−^ cells to HSV-1 infection is independent of IFN-β.

**FIG 4:**
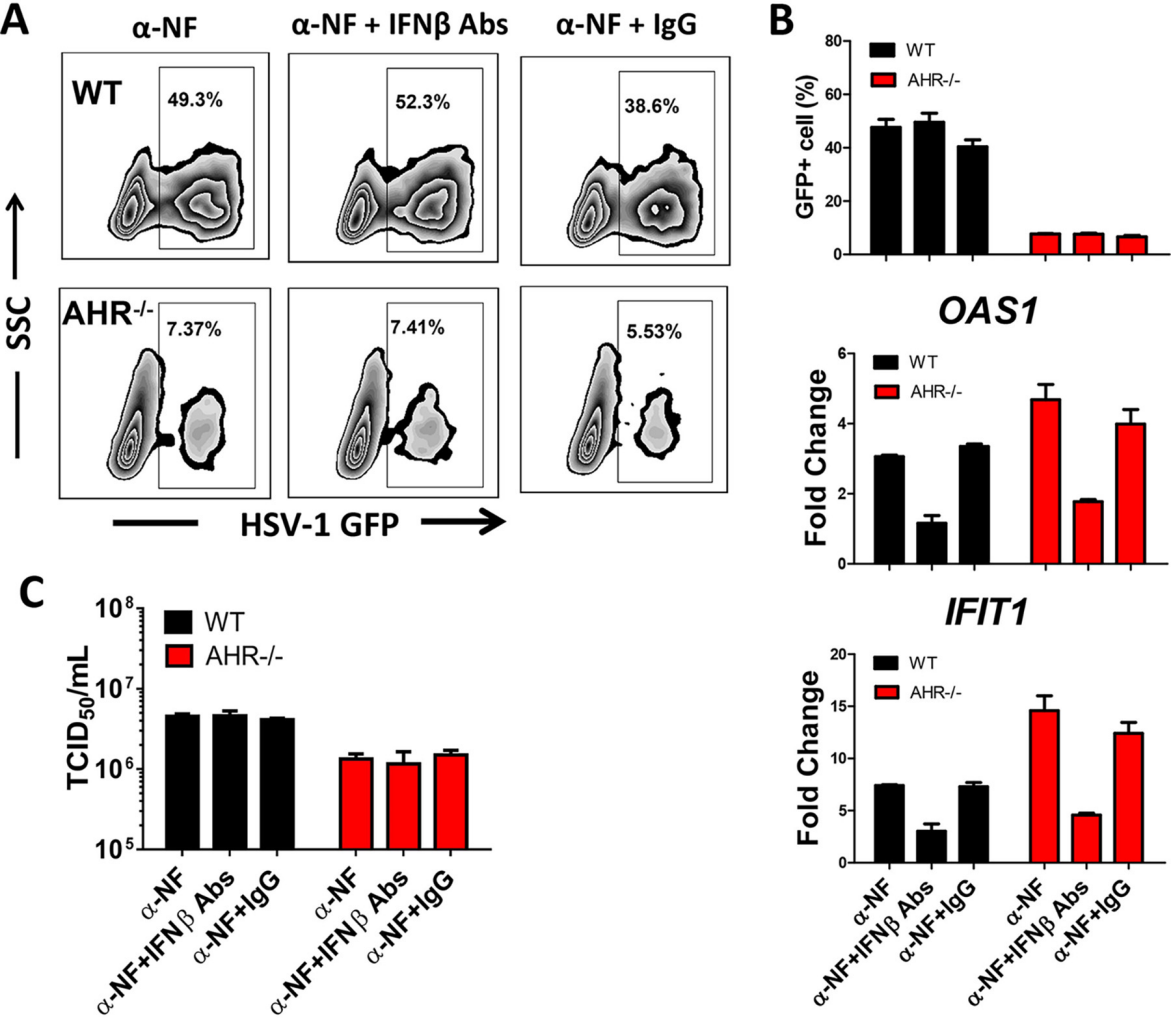
Enhanced resistance of *AHR*^−/−^ THP-1 cells to HSV-1 infection is independent of IFN-β. WT THP-1 cells and *AHR*^−/−^ THP-1 cells were pretreated with α-NF for 24 h, and IFN-β antibodies (Abs) or isotype antibody was added at the same time as HSV-1 infection for 24 h. (A) Percentages of GFP^+^ cells were analyzed by flow cytometry. (B) Percentages of GFP-positive cells are shown in a histogram. The mRNA of the IFN-inducible genes *IFIT1* and *OAS1* was measured by qPCR. (C) Viral titers in each group were determined by a TCID_50_ assay. *, *P* < 0.05; **, *P* < 0.01. Data are shown as means ± SEM.

To investigate whether the increased resistance of *AHR*^−/−^ THP-1 cells to HSV-1 infection is dependent on IFN-β, we first quantified the percentage of virally infected cells (GFP^+^) and the expression of the IFN-inducible genes *IFIT1* and *OAS1* 6 h and 24 h after infection in the presence or absence of a neutralizing antibody to human IFN-β. We observed that the addition of IFN-β neutralizing antibody did not increase the extent of HSV-1 infection in *AHR*^−/−^ cells (Fig. 4A and B), although the expression of the IFN-inducible genes *IFIT1* and *OAS1* was reduced in both WT and *AHR*^−/−^ cells upon neutralizing IFN-β (Fig. 4B; Fig. S4). Similarly, viral titers showed no statistical differences in WT and *AHR*^−/−^ cell cultures upon neutralizing IFN-β (Fig. 4C), suggesting that the enhanced resistance of *AHR*^−/−^ cells to HSV-1 replication is independent of IFN-β.

### Inhibition of AHR restricts HSV-1 replication in *STAT1*^−/−^ HFF-1 cells.

**FIG 5:**
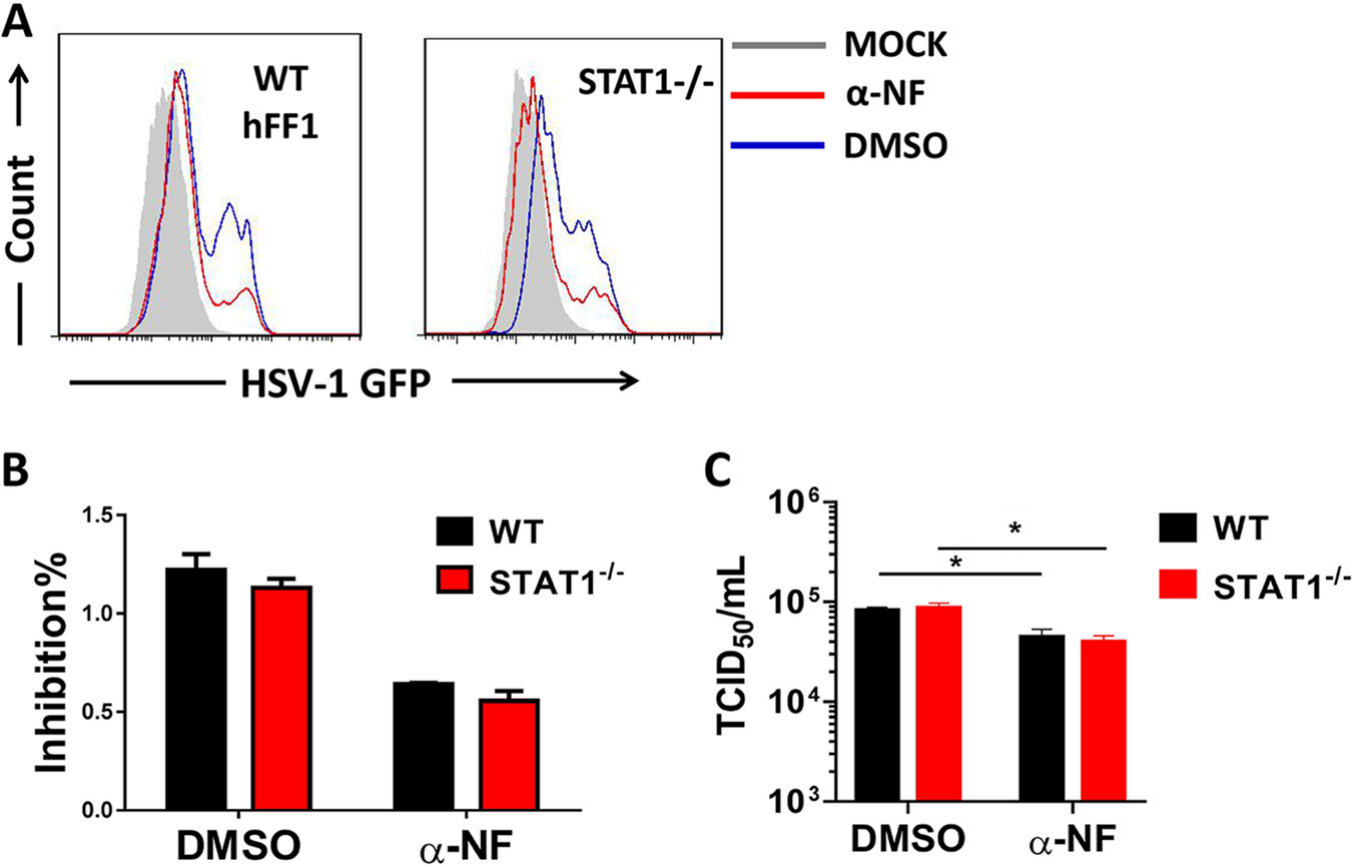
Inhibition of AHR still restricts HSV-1 infection in *STAT1*^−/−^ HFF-1 cells. α-NF- or mock-stimulated WT and *STAT1*^−/−^ HFF-1 cells were infected with GFP-expressing HSV-1 for 24 h. (A) The percentages of GFP-positive cells were determined using flow cytometry. (B) The inhibition rate was determined by the percentage of GFP^+^ cells in the DMSO- or α-NF-treated group divided by that of the untreated group. (C) Viral titers in each group were determined by a TCID_50_ assay. *, *P* < 0.05; **, *P* < 0.01. Data are shown as means ± SEM.

To formally exclude the role of type I IFN signaling in the enhanced antiviral resistance resulting from AHR deficiency, *STAT1*^−/−^ HFF-1 cells lacking type I IFN signaling were used. WT and *STAT1*^−/−^ HFF-1 cells were treated with α-NF, followed by infection with GFP-expressing HSV-1. We found that the inhibition of AHR signaling resulted in comparable reductions in viral loads in WT and *STAT1*^−/−^ HFF-1 cells (Fig. 5A and B). Similarly, HSV-1 titers determined using the TCID_50_ assay displayed similar patterns (Fig. 5C), indicating that type I IFN signaling does not contribute to the enhanced antiviral activity observed in cells treated with α-NF.

## DISCUSSION

It has been reported that AHR activation during influenza virus infection decreased mouse survival by inducing increased lung IFN-γ production by macrophages and neutrophils (18). Here, we examined the role of AHR signaling in HSV-1 infection in THP-1 cells, a human monocytic cell line commonly used to research the function of macrophages and monocytes. The above-described results in human THP-1 cells are consistent with reported research in mouse embryonic fibroblasts (MEFs) (15), suggesting that AHR deficiency enhances defense against HSV-1 infection by restraining virus replication. Although the reported results indicated that AHR-mediated signaling impairing the host antiviral defense system was associated with IFN-β production in MEFs, the question of whether increased IFN production contributes to the restriction of viral replication has not been examined directly. We found that after neutralizing IFN-β, HSV-1 titers in *AHR*^−/−^ THP-1 cultures are not affected, indicating that the antiviral effect of AHR-mediated signaling was independent of IFN-β. Type I IFNs play a critical role against viral infection involving IFN-β and various IFN-α subtypes; importantly, IFN-α and IFN-β have the common receptor IFNAR, the specific binding sites separately and different affinity with IFNAR direct to variant functions (19), IFN-α may have a key role in viral resistance *in vivo*. However, the results from our *STAT1*^−/−^ HFF-1 cells preclude the above-mentioned hypothesis based on the observation that α-NF treatment had no effect on the HSV-1 control in *STAT1*^−/−^ HFF-1 cells, which totally lack downstream type I IFN signaling.

In order to survive in host cells, HSV-1 must hijack multiple host mechanisms to evade antiviral machinery or to assist self-replication, which may explain the enhanced IFN-independent antiviral resistance of *AHR*^−/−^ THP-1 cells. Since viral genomes have a limited coding capacity, DNA/RNA viruses often hijack cellular factors to promote their proliferation in host cells. It was reported that TRIM26, an E3 ligase, was a critical host factor for hepatitis C virus (HCV) replication and contributed to host tropism (20). There is also evidence that HSV-1 hijacks cofilin to facilitate virus entry into neuronal cells (21). AHR, as a transcriptional factor of the basic helix-loop-helix-PER-ARNT-SIM (bHLH-PAS) family, also participates in regulating many genes involved in viral defense directly or indirectly. As in Zika virus (ZIKV) infection, virus-activated AHR worked as a host factor for ZIKV replication and suppressed intrinsic immunity driven by the promyelocytic leukemia (PML) protein (22). Also, AHR was identified as a key proviral factor since its expression was upregulated in first-trimester trophoblast cells infected with ZIKV (23). It also has been reported that HIV-1 infection and reactivation are positively correlated with AHR activation (24). The binding of activated AHR to the human T-cell lymphotropic virus type 1 (HTLV-1) long terminal repeat (LTR) dioxin response element (DRE) site has been shown to drive HTLV-1 plus-strand transcription, which is critical for viral reactivation and replication (25). Human CMV (HCMV) infection led to elevated levels of kynurenine, which is an endogenous AHR ligand, and knockdown or inhibition of AHR with chemicals decreased viral RNA levels and ameliorated RNA expression, which are associated with the cell cycle and blocked in CMV infection (26). Classically, interferon-stimulated genes (ISGs) that are critical antiviral factors had been believed to be dependent on type I IFN production (27). However, a recent study reported that interferon regulatory factor 3 (IRF-3) expression upregulated ISGs in an IFN-independent manner in the context of HCMV infection (28). Similar outcomes were also observed in HSV-1 or HIV infection experiments (29, 30). TCDD-inducible poly(ADP-ribose) polymerase (TIPARP), which is induced by AHR ligand, could suppress IRF-3 dimer formation (15). Indeed, it can be inferred that AHR may influence viral infection through impacting IRF-3 independent of IFN. Interestingly, gut and skin microbes regulate neurogenesis and skin barrier function via AHR signaling in mice based on mice lacking AHR having neurogenesis dysfunction and being more liable to infection (31, 32), suggesting the existence of an opposite role of this receptor when infected with different microorganisms.

Our study uncovers that an interruption of the AHR pathway results in enhanced antiviral resistance in a type I IFN-independent manner. These findings are consistent with recent studies demonstrating that AHR signaling can promote infections by several viruses, including HSV-1 (15, 18, 22, 25). Strikingly, novel research showed that an AHR inhibitor not only eliminates detrimental damage in respiratory function caused by IFN-β or IFN-γ but also ameliorates lung pathology caused by severe acute respiratory syndrome coronavirus 2 (SARS-CoV-2) in hACE2-transgenic mice (33). Taken together, our findings suggest that the local application of AHR antagonists at the accessible infected tissue site, such as skin or airway, could potentially be considered an antiviral treatment option.

### Conclusions.

Collectively, we have identified that AHR enhances HSV-1 replication.

## MATERIALS AND METHODS

### Construction of *AHR*^−/−^ THP-1 cell lines.

Lentivirus technology and CRISPR/Cas9 technology were combined to successfully prepare CRISPR/Cas9 lentivirus with the targeted AHR gene. Coinfection was done with lenti-Cas9-blasticidin (elongation factor-1α short promoter [EFS]-Cas9-Flag-blast virus), a guide RNA (gRNA) (rev response element [RRE]-U6-gRNA-EF1a-puro virus) to express Cas9, and a guide RNA targeting AHR in THP-1 cells, which were screened by blasticidin and then subjected to restricted dilution. After extended culture, the *AHR*^−/−^ monoclonal cell line was identified using immunoblotting and sequencing.

### Cell culture and viral infection.

THP-1 cells were cultured in RPMI 1640 medium supplemented with 15% fetal calf serum (FCS) and penicillin/streptomycin. *STAT1*^−/−^ human foreskin fibroblast 1 (HFF-1) cells were cultured in Dulbecco’s modified Eagle’s medium (DMEM) supplemented with 10% FCS and penicillin/streptomycin. Vero cells were cultured in DMEM containing 10% fetal bovine serum (FBS), 2 mM glutamine, 1.0 mM sodium pyruvate, and 0.1 mM nonessential amino acids. All cells were maintained at 37°C in a humidified atmosphere with 5% CO_2_. α-Naphthoflavone (α-NF) (catalog no. N5757; Sigma-Aldrich), an AHR antagonist, was used to block AHR signaling in THP-1 cells by pretreating the cells for 24 h, and the AHR ligand 2,3,7,8-tetrachlorodibenzo-*p*-dioxin (TCDD) (catalog no. NIST1614; Sigma-Aldrich) was used for stimulation. Interruption of type I IFN signaling was conducted using IFN-β antibody (catalog no. AB1431; Merck Millipore) or IFNAR antibody (catalog no. 21385-1; PBL Assay Science) with isotype antibody (catalog no. I5006; Sigma-Aldrich) as a control, and the cells were then infected with green fluorescent protein (GFP)-expressing HSV-1. GFP-expressing HSV-1 and CMV, which could be detected by flow cytometry or confocal microscopy during cellular infection, were generated as described previously (9, 34). In brief, Vero cells were transfected with cosmids and an HSV-1 or CMV amplicon plasmid that carries the *gfp* gene for several hours and then washed and incubated with packaging medium. Viruses were harvested at the indicated hours, and virus stocks were stored at −80°C for further use.

### Immunoblotting.

Cells were collected, lysed in radioimmunoprecipitation assay (RIPA) buffer with phosphatase and protease inhibitors, and centrifuged at 12,000 rpm for 15 min at 4°C. The concentration of the supernatant was determined by a bicinchoninic acid (BCA) protein assay kit (catalog no. CW0014; CoWin Biosciences). The samples were detected using Western blotting with primary antibodies recognizing AHR (catalog no. 83200S; Cell Signaling Technology) and tubulin (catalog no. 2128S; Cell Signaling Technology) for 2 h and then with secondary antibodies (catalog no. 7074P2; Cell Signaling Technology) for 1 h. The proteins were detected by using a chemiluminescent substrate (catalog no. 32109; Pierce).

### Cytokine assay.

WT and *AHR*^−/−^ THP-1 cells were infected with GFP-expressing HSV-1. After 24 h, the cell culture supernatant was collected. The concentration of IFN-β in the supernatant was measured with commercial enzyme-linked immunosorbent assay (ELISA) kits for IFN-β (catalog no. 41415; PBL Assay Science), according to the manufacturer’s instructions. Each sample was tested in triplicate, and plates were read using a microplate reader.

### Immunofluorescence and flow cytometry.

For immunofluorescence staining, after being fixed with 4% paraformaldehyde (PFA) for 30 min, HSV-1-infected cells were permeabilized with 0.5% Triton X-100 for 5 min and blocked in 3% bovine serum albumin for 30 min. Cells were then stained with 4,6-diamidino-2-phenylindole (DAPI) before mounting onto slides and imaging under a confocal microscope. On the other hand, THP-1 cells and HFF-1 cells were infected with GFP-expressing HSV-1 for 24 and/or 48 h. Cells were collected, washed with phosphate-buffered saline (PBS), resuspended in PBS, and analyzed by flow cytometry (FACSAria II; BD).

### Quantitative real-time PCR.

Total RNA was extracted using a total RNA kit (catalog no. R6834; Omega) according to the manufacturer’s instructions. The extracted RNA was reverse transcribed to cDNA. The transcript levels of the specified gene were quantified using a SYBR green detection method by real-time PCR on ABI7500 instruments (Thermo). The housekeeping gene *hGAPDH* was used as an internal control to calculate the relative expression level. All the primer sequences are available in the supplemental material.

### Virus titer determination.

The virus titer was measured in Vero cells by a TCID_50_ assay. Briefly, cells were infected with GFP-expressing HSV-1 for 1, 6, 24, and 48 h and then collected and lysed, and lysates were 10-fold serially diluted from 10^−1^ to 10^−11^. Vero cells were seeded in a 96-well plate until 70 to 90% confluent, infected with 100 μl of diluted lysates for 2 h, and maintained in DMEM containing 1% FBS. The plates were then incubated at 37°C in a humidified atmosphere with 5% CO_2_ and checked daily until cytopathic effects (CPE) had no more progress. The TCID_50_ per milliliter values were calculated by using the Reed-Muench method (35).

### Statistical analysis.

Student’s *t* test or one-way analysis of variance (ANOVA)/Newman-Keuls multiple-comparison test was used for statistical analyses to compare different conditions, data are shown as means ± standard errors of the means (SEM), and *P* values of less than 0.05 were considered significant. Data are representative of results from three biologically independent experiments. GraphPad Prism 6 software (GraphPad Software, CA, USA) was used for data analysis.
