# Supplementary material for: Targeting Aryl Hydrocarbon Receptor Signaling Enhances Type I Interferon-Independent Resistance to Herpes Simplex Virus
Source: Microbiol Spectr. 2021 Oct 20;9(2):e00473-21. doi: 10.1128/Spectrum.00473-21 (PMC8528105; doi:10.1128/Spectrum.00473-21)
Supplement: SUPPLEMENTAL FILE 1 — Supplemental material. Download Spectrum.00473-21-s0001.pdf, PDF file, 0.5 MB [file spectrum.00473-21-s0001.pdf]

Fig. S1

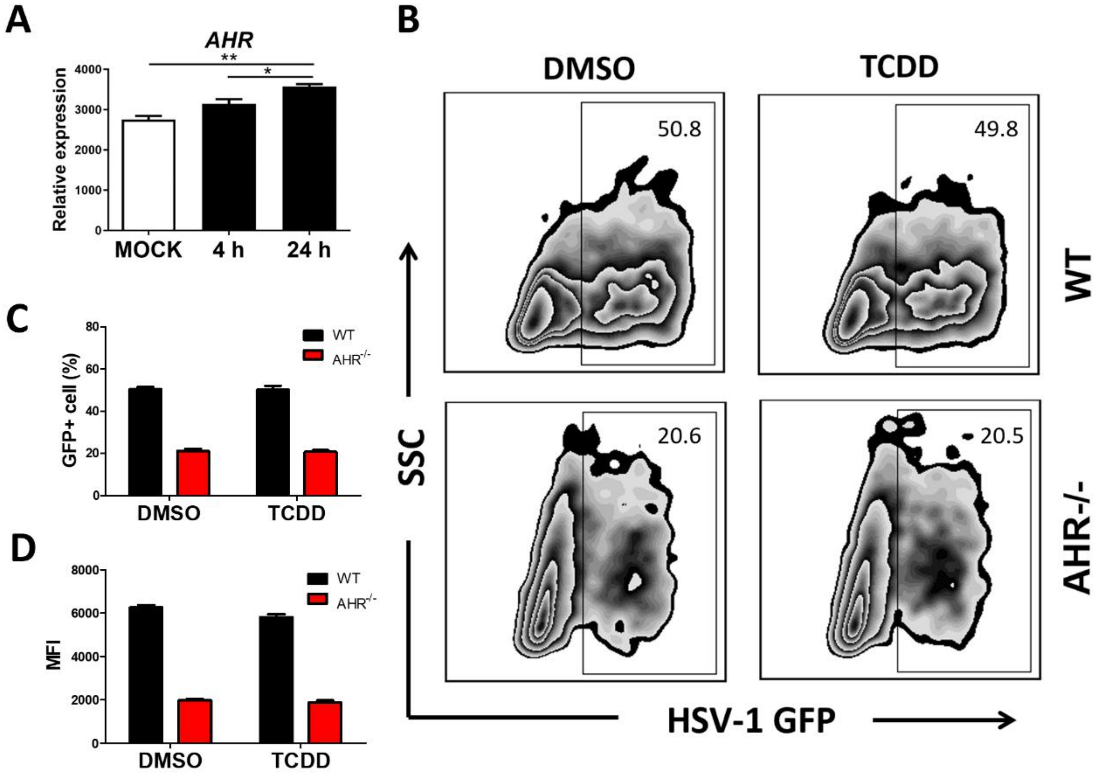

Fig. S1. AHR ligand TCDD couldn't promote HSV-1 infection.

WT and *AHR*$^{-/-}$ THP-1 cells were pretreated with AHR ligand TCDD or DMSO (as negative control) and then infected with HSV-1 GFP for 24h. The mRNA expression level of AHR in THP-1 cells infected with HSV-1 at indicated time point (A), Flowcytometry was used to show the proportion of auto-fluorescent GFP (B), corresponding statistic percentage of GFP positive cell (C) and mean fluorescence intensity (MFI) of GFP (D).

Fig. S2

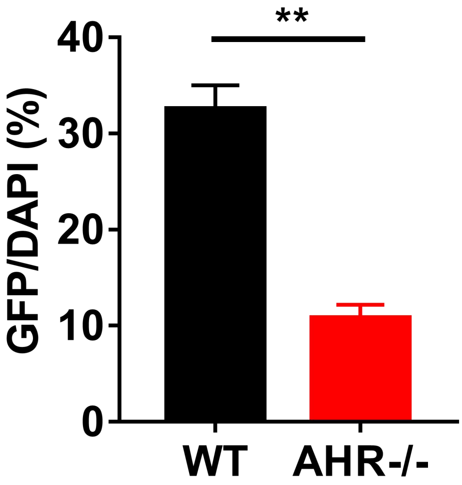

Fig S2. Quantification of HSV-1 GFP+ cells using confocal microscopy. WT and *AHR*$^{-/-}$ THP-1 cells were infected with GFP-expressing HSV-1 virus. The percentage of GFP positive cells was quantified by confocal microscopy at 24h.

Fig. S3

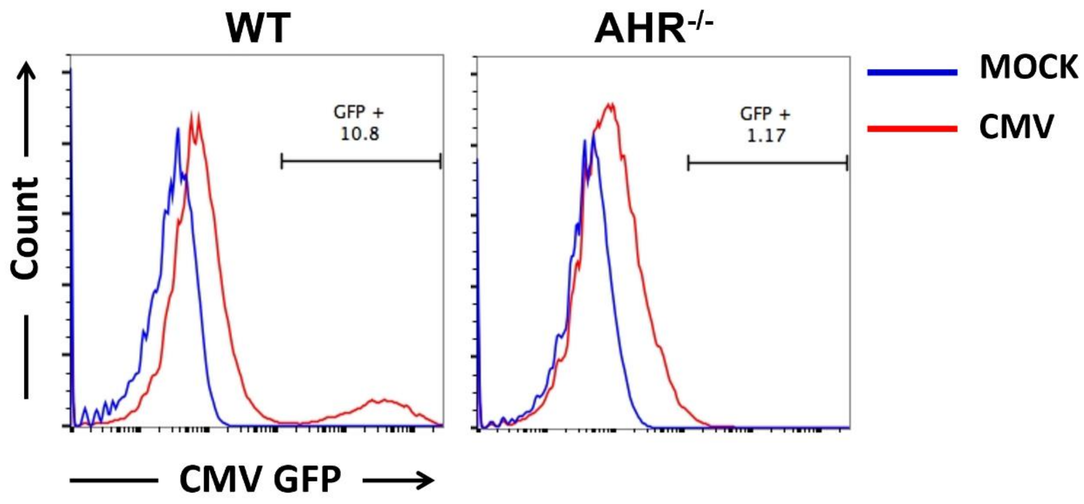

Fig. S3. *AHR^{-/-}* THP-1 cells resist against CMV infection. WT and *AHR^{-/-}* THP-1 cells were infected by cytomegalovirus (CMV) tagged with GFP for 24h and CMV-GFP intensity were determined by Flow cytometry.

Fig. S4

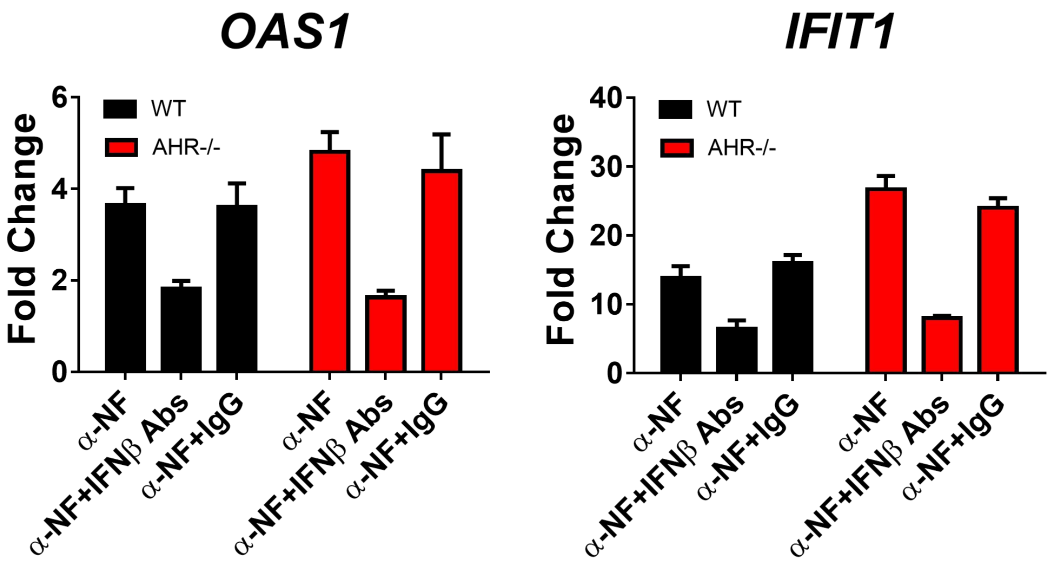

Fig S4. ISGs expression after neutralizing IFN-β at 6h post-infection. WT and AHR-/- THP-1 cells were pretreated with α-NF for 24h and IFN-β antibody or isotype antibody were added at the same time as HSV-1 infection. The mRNA expression of IFN-inducible genes IFIT1 and OAS1 was measured by qPCR at 6h after HSV-1 infection.

Table S1. qRT-PCR primer sequences

primers	sequences
AHR- gRNA-F	CACCGAAGTCGGTCTCTATGCCGCT
AHR- gRNA-R	AAACAGCGGCATAGAGACCGACTT
hCYP1B1- F	ACGTACCGGCCACTATCACT
hCYP1B1- R	CTCCCCACGACCTGATCCA
hAHR-F	ACATCACCTACGCCAGTCG
hAHR-R	CGCTTGGAAGGATTTGACTTGA
hARNT-F	CTGCCAACCCCGAAATGACAT
hARNT-R	CGCCGCTTAATAGCCCTCTG
hIFN-β-F	GTCACTGTGCCTGGACCATAG
hIFN-β-R	GTTTCGGAGGTAACCTGTAAGTC
hOAS1-F	TGTCCAAGGTGGTAAAGGGTG
hOAS1-R	CCGGCGATTTAACTGATCCTG
hIFIT1- F	GCGCTGGGTATGCGATCTC
hIFIT1- R	CAGCCTGCCTTAGGGGAAG
hGAPDH-F	GCACCGTCAAGGCTGAGAAC
hGAPDH-R	TGGTGAAGACGCCAGTGGA
